# Chicken Egg Proteins and Derived Peptides with Antioxidant Properties

**DOI:** 10.3390/foods9060735

**Published:** 2020-06-03

**Authors:** Sara Benedé, Elena Molina

**Affiliations:** Instituto de Investigación en Ciencias de la Alimentación (CIAL, CSIC-UAM), 28049 Madrid, Spain; e.molina@csic.es

**Keywords:** egg white, egg yolk, antioxidant peptides

## Abstract

In addition to their high nutritional value, some chicken egg proteins and derivatives such as protein hydrolysates, peptides and amino acids show antioxidant properties which make them prominent candidates for the development of functional foods, drawing attention to both the food and biopharmaceutical industries. This review summarizes current knowledge on antioxidant activity of chicken egg proteins and their derived peptides. Some egg proteins such as ovalbumin, ovotransferrin and lysozyme from egg white or phosvitin from yolk have shown antioxidant properties, although derived peptides have higher bioactive potential. The main process for obtaining egg bioactive peptides is enzymatic hydrolysis of its proteins using enzymes and/or processing technologies such as heating, sonication or high-intensity-pulsed electric field. Different in vitro assays such as determination of reducing power, DPPH and ABTS radical-scavenging activity tests or oxygen radical absorbance capacity assay have been used to evaluate the diverse antioxidant mechanisms of proteins and peptides. Similarly, different cell lines and animal models including zebrafish, mice and rats have also been used. In summary, this review collects all the knowledge described so far regarding egg proteins and derived peptides with antioxidant functions.

## 1. Introduction

Eggs are not usually considered as antioxidant foods, however, many of their compounds such as vitamin E and A, selenium, phospholipids and carotenoids exhibit antioxidant properties [1]. In addition to their high nutritional value, chicken egg proteins and related ingredients (protein hydrolysates, peptides and amino acids) show several biological activities, including antioxidant activity, and therefore their use as functional and nutritional ingredients in food products has increased in recent years, drawing the attention of both the food and biopharmaceutical industries [2]. Moreover, natural antioxidants are considered safer for consumers than synthetic antioxidants and therefore there is a growing interest in them.

Egg white is mainly composed of protein (11%), being ovalbumin the most abundant (54%) followed by ovotransferrin (12%), ovomucoid (11%), lysozyme (3.5%), and ovomucin (3.5%). Besides, other minor proteins such as avidin, cystatin, ovomacroglobulin, ovoflavoprotein, ovoglycoprotein and ovoinhibitor have also been identified [3]. The main components of the yolk are lipids (31–35%) although it also has 15–17% of proteins including lipovitellins (36%), livetins (38%), phosvitin (8%), and low-density lipoproteins (17%) [4]. Egg yolk is covered with the vitelline membrane which separates it from the egg white and it is also a good source of proteins, composed mostly of protein fibers [5].

Some of these proteins have antioxidant properties by themselves but it has been demonstrated that peptides derived from them, usual fragments of 2–20 amino acid residues, have the higher bioactive potential [6]. The hypothesis that arises to explain this fact is that small peptides have increased accessibility of the functional side chain (R-group) to the reactive species and the electron-dense peptide bonds and therefore they can exert their antioxidant function more easily [7]. However, not only peptide length is associated with the activity of antioxidant peptides and amino acid composition seems to have an important role. The sulfur-containing amino acids such as cysteine and methionine are prone to oxidation due to their S-groups that form stable oxidation products by reacting with reactive species. Acidic amino acids such as glutamine and asparagine and the hydrophobic proline, alanine, cysteine, valine, methionine, isoleucine, leucine, tyrosine, phenylalanine and tryptophan amino acids have also a strong positive effect on antioxidant activity [7]. Functional peptides are not active within the sequence of the parent protein molecule and can be released by in vivo or in vitro processes [8]. The main procedure for obtaining bioactive peptides from food products is enzymatic hydrolysis of proteins orchestrated with the use of various enzymes of microbial, plant or animal origin [9], although chemical hydrolysis [10] or processing technologies such as heat or high-intensity pulsed electric field treatments can also be applied [11,12].

Antioxidant peptides from eggs can inhibit oxidative stress, which plays an important role in human health, and in food systems, and increase the quality and the shelf life of products. Moreover, antioxidant peptides prevent oxidative damages through multiple pathways such as free radical scavenging, chelating pro-oxidative transition metal ions, inactivation of reactive oxygen species and reducing hydroperoxides [9,13,14] and, therefore, there is not a single antioxidant test model to evaluate their activity. In practice, several in vitro assays have been developed to quantify antioxidant activities and can be classified into two types; hydrogen atom or electron transfer reaction-based assays [15]. Hydrogen atom transfer reaction-based assays quantify the hydrogen atom donating ability of the antioxidant compound resulting in a kinetic curve, while the electron transfer reaction-based assays measure the reducing capacity of the antioxidant compound, resulting in a color shift that can be measured by the change in absorbance [16]. The 1,1-diphenyl-2-picrylhydrazyl (DPPH) radical scavenging activity and reducing power assays are the most used in studies on the antioxidant capacity of egg derivatives [17]. Among the hydrogen atom transfer-based method most commonly used methods to measure the antioxidant capacity of egg-derived peptides is oxygen radical absorbance capacity (ORAC) which consist in a fluorescent compound, such as fluorescein, that is damaged by free radicals and subsequently loses its fluorescence, but when antioxidants are present the loss of fluorescence is inhibited [18]. The 2, 2-azinobis (3-ethyl benzothiazoline-6-sulfonic acid) diamonium salt (ABTS) radical scavenging method and lipid peroxidation inhibition assay are also included in this category, among others [17,19]. Despite the numerous *in vitro* studies performed to evaluate the antioxidant capacity of egg-derived hydrolysates or peptides, their commercial application is delayed due to the lack of scalable production processes, the few digestibility and bioavailability studies as well as animal studies available and the absence of clinical trials that probe their potential health benefits. In this work, current knowledge on antioxidant activity of chicken egg proteins, the main strategies to obtain antioxidant peptides from them and the identified peptides are summarized.

## 2. Antioxidant Activity of Chicken Egg Proteins

### 2.1. Ovalbumin

Ovalbumin is the most abundant protein in egg white. It has a molecular mass of 45 kDa and is compiled of 385 amino acids. It contains one disulfide bond and four free sulphydryl groups, which can play a role in redox regulation, acting as metal chelators [20]. Several studies have described an increased antioxidant capacity of ovalbumin after covalent binding. Glycosylation of ovalbumin with glucose under heat moisture treatment [21] or microwave heating [22] increased its antioxidant activity measured by determination of DPPH radical-scavenging activity and Trolox equivalent antioxidant capacity assay. Glycation with mannose by ultrasound technology [23], maltose by heat treatment [21] or covalent binding with dextran or galactomannan by a controlled Maillard reaction [24] also exhibited higher antioxidant activity than native ovalbumin. In addition, the raising of antioxidant activity has also been identified when ovalbumin was combined with the polyphenol rutin [25] or the mineral selenite [26] which was attributed to the formation of a molten globule conformation of the protein, increasing its surface hydrophobicity and therefore its solubility.

### 2.2. Ovotransferrin

Ovotransferrin is composed of 686 amino acids and has a molecular mass of 77.90 kDa. It is folded into two globular lobes with an iron-binding site and interconnected by an alpha-helix of nine amino acidic residues. Fifteen disulfide bridges stabilize the structure [27]. In addition to antimicrobial activity, antioxidant properties have also been attributed to ovotransferrin [10]. Binding of ovotransferrin with metals such as iron, magnesium and copper [28], conjugation with small molecules such as catechin [29] or autoclaved treatment improved its antioxidant activity [30]. 

### 2.3. Lysozyme

Lysozyme contains 129 amino acids, presents a molecular mass of 14.3 kDa and four disulfide bridges. It inhibits reactive oxygen species generation [31] and like other egg white proteins, its antioxidant properties increased after conjugation with other compounds such as polysaccharides [32]. Conjugation of lysozyme with guar gum, a hydrophilic polysaccharide extracted from the seeds of *Cyamopsis tetragonolobus* increased its antioxidant properties from 2% to 35% of inhibition of DPPH [33]. The alkaline pH used for the preparation of the conjugate opened the globular structure of the protein, causing electron-donating amino acid residues to get more exposed and therefore increasing lysozyme reducing power. The same effect was observed when xanthan gum, an anionic extracellular polysaccharide secreted by the microorganism *Xanthomonas campestris* was used for the conjugation [34].

### 2.4. Cystatin

Cystatin is a small protein of approximately 13 kDa molecular weight which contains two disulfide bonds. It is been shown that it modulates the synthesis and release of nitric oxide production in murine macrophages and thereby plays a role in cellular antioxidant pathways [35]. Optimum levels of nitric oxide are essential for the regulation of specific cellular antioxidant pathways. In addition, cystatin could protect brain neurons from oxidative damage [36].

### 2.5. Phosvitin

Phosvitin is the major protein component in egg yolk. It has a sequence of 216 amino acid residues that contains 123 serine residues of which most are phosphorylated. Phosvitin has high chelating power for cations which has led to several studies that prove its great antioxidant properties [37,38]. Moreover, free aromatic amino acids such as tryptophan and tyrosine in egg yolk were found to be largely responsible for the antioxidant properties of egg yolk [39].

## 3. Production of Antioxidant Peptides from Chicken Egg Proteins

Bioactive peptides from eggs have been mainly produced from egg white proteins, although egg yolk has also recently been used as a new source of functional peptides, as well as other egg components such as eggshell or vitelline membrane. The whole egg white and yolk or a single protein can be used as starting material to produce bioactive peptides. The main procedure used to obtain peptides has been hydrolysis with food-grade proteolytic enzymes from animal, plant or bacterial origin [9]. Commercial enzymes are preferred over naturally occurring ones because their specific characteristics such as optimal pH, temperature or cleavage site are well defined [40]. Normally, one or several proteases are used to obtain proteins hydrolysate [41] being pepsin, trypsin, alcalase and papain some of the most popular enzymes used [12,42,43,44]. Alternatively, non-commercial enzymes have also applied to produce functional egg peptides to reduce the cost of hydrolysis [41,45].

Modification of egg proteins previously to the hydrolysis process results in protein conjugates that lead to increase radical scavenging properties [46] and therefore several physical methods such as high-intensity pulsed electric field [12], sonication [11], heat [12] and high pressure [47] treatments have been applied to increase enzymatic digestibility. The advantage of hydrolysis is that it is easy to scale up but there are numerous variables involved in the production and purification of antioxidant peptides and optimization is not taken into account in most of the published literature.

Once the hydrolysate has been obtained, the use of techniques such as ultrafiltration or liquid chromatography allows it to be separated into different fractions that can be evaluated to determine their antioxidant potential. The techniques most widely used for the measurement of antioxidant capacity are in vitro colorimetric assays in which samples compete with substrates for the radicals and inhibits or restrict the substrate oxidation [48]. After the identification of hydrolysate fractions with antioxidant properties, the generated peptides can be identified by mass spectrometry and chemically synthesized for validation.

### 3.1. Hydrolysis of Egg White

Pepsin is one of the most used enzymes to obtain egg white hydrolysates. Dávalos et al. described that 3-h proteolysis of crude egg white at pH 2 and 37 °C with an enzyme to substrate ratio of 1/100 increased the radical scavenging activity by approximately threefold compared to untreated crude egg white, being the fraction lower than 3 kDa the most active, probably because of the higher accessibility of small peptides to the redox reaction system [49]. They identified four peptides from ovalbumin with higher radical scavenging activity than that of Trolox and with lipid peroxidation inhibition ability (Table 1). Their increased antioxidant activity was imputed to the presence of tyrosine at the N terminus as the presence of a hydroxyl group in the tyrosine aromatic structure allows it to break the antioxidant chain by a hydrogen atom transfer mechanism [50]. 

In addition, the presence of methionine in the peptide structure could also be conducive to the increase of the antioxidant activity because cyclic oxidation and reduction of methionine is an important antioxidant mechanism [59]. The same 3-h protocol was used to produce egg white hydrolysate with pepsin and examined the effects of its long-term consumption on spontaneously hypertensive rats, which make not only an accepted animal model for human hypertension, but also for oxidative stress because lipid peroxidation directly damages cell membranes and increases the levels of vasoconstrictor hormones such as angiotensin and endothelin which directly induce hypertension [60]. Pepsin hydrolysate was effective in increasing the radical-scavenging capacity of the plasma and decreasing the malondialdehyde concentration, a biomarker of oxidative damage, in the aorta [61]. In addition, pepsin egg white hydrolysate has also been administrated to an experimental model of obesity using Zucker fatty rats. Obesity is associated with abnormal production of proinflammatory mediators by the fat tissue and the pepsin hydrolysate also reduced levels of plasma malondialdehyde [62].

In accordance with Dávalos et al., other study probed that DPPH, hydroxyl and superoxide anion radical-scavenging activities of the hydrolysates obtained with pepsin depend on the molecular weight of the generated peptides, being the fraction with 2–5 kDa peptides the one with stronger antioxidant activity [63], although it is difficult a precise comparison because of the different hydrolysis conditions used (Table 2). A wide screening of the conditions required to obtain the pepsin hydrolysates with the strongest antioxidant capacity was done by Lin et al. which determined that 4.56% of egg white as starting material with an enzyme to substrate ratio of 1.58% at pH 1.99 and 37 °C for 1 h were the optimal conditions, although they do not specify the activity of the pepsin used and a pre-treatment of 10 min at 90 °C was applied to denature egg white proteins [12].

Other studies have also probed the antioxidant capacity of hydrolyzed egg white with pepsin using other methods such as Ferric Reducing Antioxidant Power (FRAP) assay [43] or measurement of oxidative stress inhibitory activity in cell lines such as in the study Garcés-Rimón et al., where they observed a dose-dependent inhibition of reactive oxygen species production in a macrophage cell line after the treatment with an egg white hydrolysate obtained with pepsin [64].

Trypsin is another common enzyme to obtain bioactive peptides with antioxidant functions [43,58,65]. The optimal enzymatic parameters that have been described are 4.93% of egg white as the starting material with an enzyme to substrate ratio of 1.61% at pH 9.05 and 37 °C for 1 h [12]. Similarly, the optimal conditions of egg white hydrolysis with alcalase have been described as 5% of egg white protein powder as the starting material with an enzyme-to-substrate ratio of 3% at pH 11 and 50 °C for 3 h, being the hydrolysate fraction containing peptides with a molecular mass lower than 1 kDa the one with the highest antioxidant activity [66]. Similar results using the same hydrolysis protocol were reported later proving that antioxidant activity of peptides is intimately connected to their molecular weight [57]. Furthermore, other studies have also reported increased antioxidant activity of egg white hydrolysate with alcalase, although using different hydrolysis conditions [43,67,68,69].

Antioxidant peptides obtain from hydrolysis of egg white with alcalase have been identified by several authors. Yu et al. identified 11 peptides (Table 1) with DPPH radical-scavenging activity [51], and Liu et al. identified 4 peptides (Table 1) and in addition, observed that antioxidant capacity was restricted by the amino acid composition of peptides indicating that Leucine, aspartic acid, serine, glutamic acid and lysine could play an important role [57]. Moreover, a recent study that identifies four more peptides (Table 1), reported that valine at the N-terminus is useful to increases the antioxidant activity of peptides [44]. Furthermore, this study used the peptide VYLPR to investigate the antioxidant mechanism on HEK-293 cells. They observe that VYLPR could inhibit lipid peroxidation, contribute to cell membrane integrity, prevent intracellular lactate dehydrogenase activity, which is increased under the condition of oxidative stress, reduce the oxidative biomarker malondialdehyde, and improve the activity well known antioxidant enzymes such as superoxide dismutase and glutathione peroxidase [44]. 

Hydrolysis of egg white by papain can also produce hydrolysates with the ability to quench the superoxide anion and hydroxyl radicals, prevent lipid peroxidation and show reducing power [58]. Two peptides, YLGAK and GGLEPINFN (Table 1) showed strong antioxidant activity in DPPH radical scavenging and lipid peroxidation inhibition tests [58]. Other commercial enzymes have also been used to obtain egg white hydrolysates with antioxidant properties. Neutrase, protamex, collupulin, ficin, flavourzyme, protease M and protease P have been successfully used to obtain egg white hydrolysates with high radical scavenging activity [1,65,67]. In fact, four peptides (Table 1), obtained after hydrolysis of egg white proteins with protease P and with high oxygen radical absorbance capacity have been identified [1].

In addition to commercial enzymes, there is growing attention in the finding of microbial proteases generated by fermentation procedures. In this line, some recent studies have purified fungal proteases and subsequently used to hydrolyze egg white, resulting in hydrolysates with high antioxidant activity. Garcés-Rimón et al. used flavourzyme 1000 L and peptidase 433 P to hydrolyze commercial pasteurized egg white and obtained hydrolysates with a high oxygen radical absorbance capacity. Moreover, these results were confirmed in a macrophage cell line where they showed a dose-dependent inhibition of reactive oxygen species generation [64]. A new enzyme produced by *Aspergillus avenaceus* URM 6706 has been used in the hydrolysis of egg white at pH 10.0 and 50 °C and a positive correlation between the in vitro antioxidant activity and the degree of hydrolysis has been observed [41]. Similar results were obtained when fungal proteases obtained from *Eupenicillium javanicum* and *Myceliophthora thermophile* were used [45].

A combination of different enzymes can also be used to produce egg white hydrolysates. Different combinations of pepsin, chymotrypsin and Alcalase 2.4 L to performed double enzyme hydrolysis of egg white could produce highly antioxidative peptides [43]. The hydrolysate with higher antioxidant activity was obtained with the combined use of pepsin and chymotrypsin because, due to their cutting sites, a hydrolysate with more aromatic aminoacid residues was obtained and these aromatic residues can quench the free radicals by electron transfer [43]. 

Due to the fact that smaller antioxidant peptides could exert better biological effects [70], technological treatments have been applied before or after enzymatic hydrolysis of egg white with the purpose to increase the decomposition of proteins. The most commonly used treatment is heat to denature proteins prior to hydrolysis and facilitate access to enzymes [12,43,51]. Microwave, high-pressure and ultrasound pre-treatments of egg white have also been applied to obtain hydrolysates with higher antioxidant activity after pepsin, trypsin and alcalase hydrolysis, respectively [22,71,72]. Other treatments such as pulsed electric fields can also be applied after the hydrolysis process. Lin et al. used this technology to treat the fraction containing peptides with a molecular mass lower than 1 kDa of an egg white hydrolysate obtained with alcalase [66]. As a result, the antioxidant activity of the treated peptide fraction was increased due to the higher number of small peptides and the exposure of histidine, proline, cysteine, tyrosine, tryptophan, phenylalanine, and methionine residues.

Proteolytic degradation of egg white during the digestion process can also promote the formation of antioxidant peptides as it has been proven in several studies using simulated gastrointestinal digestions assays [73,74]. Measurement of antioxidant capacity of cooked eggs after simulated gastrointestinal digestion indicated that although the cooking of eggs reduced their antioxidant activity, the generated peptides showed higher antioxidant activity, being three peptides derived from ovalbumin (DSTRTQ, DVYSF and ESKPV) identified with antioxidant activity in a smooth muscle cell line [74]. This study used a dynamic system to mimic conditions in the gastrointestinal tract, comprising four compartments that represent the stomach, duodenum, jejunum and ileum. Stability during digestion is important to ensure the bioavailability of bioactive peptides and obtain the desired activity when tested in vivo [75]. Similarly, Jahandideh et al., also observed a reduction in tissue oxidative stress in spontaneously hypertensive rats after the administration of fried egg white previously digested in a simulated gastro-intestinal digestion system using pepsin and pancreatin [76]. However, it should be noticed that more research is needed to determine the effect of digestion on antioxidant peptides release from egg proteins because differences have been observed when in vivo research is performed. As an example, fried whole egg previously digested with commercial pepsin and pancreatin reduced tissue oxidative stress in spontaneously hypertensive rats, but the same effect was not observed when non-hydrolyzed fried whole egg was administrated to the rat despite it also underwent digestion in the digestive tract of the animals [76]. These opposite results could be attributed to differences in the origin of enzymes, time of hydrolysis, pH or temperature of hydrolysis, among others, and should be taken into account if commercially production of these peptides is desired to avoid scaling-up issues.

### 3.2. Hydrolysis of Egg Yolk

Egg yolk is also a rich generator of antioxidants due to the presence of free aromatic amino acids, being tryptophan and tyrosine two of the main contributors to the antioxidant properties of egg yolk [39]. In addition, as with egg white, antioxidant peptides can be obtained after enzymatic hydrolysis of egg yolk. Several commonly used enzymes such as pepsin, trypsin and chymotrypsin have been effective to produce egg yolk hydrolysates with antioxidant activity [77,78,79] and with the capacity to protect DNA against oxidative damage induced by peroxide [80]. The hydrolysis of defatted egg yolk with pepsin followed by pacreatin could be used to reduce stress oxidative in spontaneously hypertensive rats [76]. Other studies have identified the sequence of several egg yolk peptides derived from proteins hydrolyzsated with pepsin. Yousr and Howell, identified three peptide sequences (WYGPD, KLSDW and KGLWE) with the capacity to inhibit the peroxides and thiobarbituric acid reactive molecules in an oxidizing linoleic acid model system [81], being the superoxide anion and hydroxyl radicals scavenging and ferrous chelation the antioxidant mechanisms involved, although hydrophobic amino acids such as tyrosine and tryptophan in identified sequences could also influence [81]. Hydrolysis of egg yolk with pepsin gave rise to four peptides from Apolipoprotein B (YINQMPQKSRE; YINQMPQKSREA), Vitellogenin-2 (VTGRFAGHPAAQ) and Apovitellenin-1 (YIEAVNKVSPRAGQF) with in vitro antioxidant activity [82]. A combination of high hydrostatic pressure treatment and enzymatic hydrolysis with alcalase, elastase, savinase, thermolysin or trypsin has also been applied successfully to produce peptides derived from phosvitin with higher antioxidant activity than the native protein [83]. 

Phosvitin phosphopeptides obtain after hydrolysis of egg yolk with trypsin followed by an alkaline dephosphorylation of phosvitin were effective to reduce oxidative stress in an in vitro system of human intestinal epithelial cells [84,85]. In addition, IL-8 released after treatment of cells with H_2_O_2_ was reduced [86]. The authors attributed the strong antioxidant activity of phosvitin phosphopeptides to their rich amino acid composition in histidine, methionine and tyrosine rather than the presence of phosphorylserine ligands [87]. Phosvitin phosphopeptides have also been obtained using alcalase, being able to reduce in vitro oxidative stress by up-regulating glutathione synthesis and antioxidant enzyme activities [88]. Moreover, this activity is maintained after gastrointestinal digestion and can promote the antioxidant capacity of enzymes such as catalase and glutathione S-transferase, and reduced protein and lipid oxidation in the intestine of a porcine model of oxidative stress [89]. Alcalase was also used by Park et al., to hydrolyze egg yolk protein, obtained as a secondary product after purification of lecithin, and identify two antioxidant peptides (LMSYMWSTSM and LELHKLRSSHWFSRR). The activity of these peptides was attributed to the presence of leucine at their N-terminal positions [90].

Hydrolysis of egg yolk protein with a serine proteinase from Asian pumpkin pulp for 4 h allowed the production of a hydrolyzate with DPPH free radical scavenging capacity [91], as well as the identification of four peptides (RASDPLLSV, RNDDLNYIQ, LAPSLPGKPKPD and AGTTCLFTPLALPYDYSH) with in vitro antioxidant activity analyzed by DPPH scavenging activity, ferric reducing ability and ferrous ion-chelating activity [9]. The use of other enzymes such as serine protease from yeast has also allowed the identification of QSLVSVPGMS peptide that exhibits a high in vitro DPPH free radical scavenging activity [92]. 

Other enzymes from the microbial origin such as neutrase, thermolysin and pronase, among others, have also been used to produce hydrolysates with in vitro DPPH scavenging and chelating iron activity [77,93,94]. Sakanaka et al. reported that egg yolk protein hydrolysates, obtained with the action of proteinase from *Bacillus* sp., display antioxidant activities in a linoleic acid oxidation system [77]. They proved its antioxidant capacity on cookies containing linoleic acid and, therefore, was proposed as a natural antioxidant for avoiding the oxidation of polyunsaturated fatty acids in food products. In addition, egg yolk peptides have been also useful to inhibit lipid oxidation in other food matrices such as beef or tuna muscle homogenates [93]. In fact, as a natural protein derived from animal products, phosvitin and its derived peptides have been proposed to be used as antioxidant in meat products [95].

### 3.3. Hydrolysis of Other Egg Components

Vitelline membrane, a multilayered structure that surrounds the egg yolk separating the yolk and white, is composed of about 87% protein. Its DPPH scavenging, superoxide radical scavenging and iron-chelating activities have been proven in in vitro studies. Moreover, the antioxidant capacity of the vitelline membrane was improved after enzymatic hydrolysis with alcalase, flavourzyme or trypsin [13]. Hydrolysates from eggshell membrane proteins have been obtained using alcalase and protease S [96] with the potential to suppress lipid and protein oxidation against oxidative stress damage induced by H_2_O_2_ in Caco-2 cells. The mechanisms involved in this effect were the elevation of antioxidant enzyme activities and cellular levels of GSH, a cellular endogenous antioxidant, via up-regulation of its mRNA expression. In addition, these hydrolysates increased γ-GCS activity, which catalyzes GSH synthesis from glutamate, cysteine and glycine [97]. 

## 4. Peptides from Individual Egg White Proteins

Foods contain many naturally occurring compounds [98] that can interact with the proteins in the matrix, affecting the type of peptides generated upon hydrolysis and should be considered during the production process of antioxidant peptides [75]. Despite this fact, functional peptides have been obtained from individual egg white proteins in many studies. 

### 4.1. Ovotransferrin

After digestion of ovotransferrin by thermolysin and pepsin, the resultant hydrolysate showed higher oxygen radical absorbance value than the native protein. The peptide IRW, derived from hydrolysis, exhibited a high oxygen radical-scavenging effect, which might be attributed to the presence of tryptophan [52]. Moreover, two tetrapeptides (WNIP and GWNI) with high antioxidant activity have been identified within the digests of ovotransferrin with thermolysin. The motif of WNI seemed to be responsible for the high antioxidant capacity because amino acid residues coupled to either the N or C terminus of both peptides reduced their antioxidant capacity [11]. In addition, the peptide GWNI has shown the capacity to reduce reactive oxygen species generation when it was tested in endothelial cells [99]. In contrast, other antioxidant peptides previously identified from ovotransferrin using the oxygen radical absorbance capacity did not exhibit any antioxidant activity in cells, showing the deficiencies of cell-free in vitro methods for antioxidant studies and highlighting the need to use more biological systems such as culture cells to evaluate antioxidant peptides [99]. Hydrolyzates derived from ovotransferrin obtained with HCl at pH 2.5 or other different enzymes such as protamex, alkalase, trypsin, neutrase, flavorzyme, maxazyme, collupulin, protex, promod 278, and alpha-chymotrypsin showing higher superoxide anion scavenging activity and oxygen radical absorbance capacity than intact protein and demonstrating preventive effects against the oxidative stress-induced DNA damage in human leukocytes [10].

### 4.2. Lysozyme

Lysozyme hydrolyzed with alcalase showed similar oxygen radical absorbance activity than the synthetic antioxidants hidroxibutilanisol and butilhidroxitolueno in foods [100]. Lysozyme hydrolyzed with papain, trypsin or a combination of the two enzymes showed high DPPH scavenging activity and the peptide NTDGSTDYGILQINSR, resulting from the double hydrolysis, was identified as responsible of the hydrolysate antioxidant capacity [53]. Gastrointestinal digestion of lysozyme may also induce the generation of antioxidant peptides. Lysozyme subjected to simulated physiological digestion using pepsin, trypsin and alpha-chymotrypsin was found to have potent antioxidant capacity determined by DPPH radical scavenging and reducing power assays. In addition, three peptides with strong antioxidant capacity were identified as products of digestion [54]. Hydrolysis of lysozyme with pepsin can give rise to antioxidant peptides. Carrillo et al. identified five positively charged peptides (Table 1) with high oxygen radical absorbance capacity in vitro. They were one step further and confirmed the results in a Zebrafish larvae model testing oxidative stress by measuring the inhibition of lipid peroxidation [55]. For this, they incubated larvae with lysozyme peptides and initiated lipid peroxidation by adding H_2_O_2._ After incubation during 8 h at 28 °C, the Zebrafish larvae were homogenized and measured in a spectrophotometer. The drop of absorbance indicated an elevation of antioxidant activity.

### 4.3. Ovalbumin

Ovalbumin hydrolysate obtained by hydrolysis with pepsin was also tested in vivo using aged mice. The hydrolysate significantly decreased malondialdehyde content in the serum and liver of mice, proving its antioxidative activity [101]. Moreover, hydrolyzing ovalbumin with different combinations of enzymes such as pepsin and papain, pepsin and alcalase, alcalase and trypsin, and alpha-chymotrypsin were also effective in producing peptides with antioxidant capability as well as strong iron and copper-binding capacity [102].

### 4.4. Other Egg Proteins

Several enzymes alone such as pepsin and alcalase or in combination such as alcalase and trypsin or alcalase and trypsin have also been used to obtain ovomucoid hydrolysates, all of them showing strong antioxidant activity analyzed by the thiobarbituric acid reactive substances method [103]. Ovomucin hydrolysates obtained by the action of trypsin, papain or alcalase have also antioxidant activity. In addition, heating ovomucin under alkaline conditions gave rise peptides with strong iron-binding and antioxidant activities [104]. In addition, antioxidant peptide derived from the ultrafiltrate of ovomucin hydrolysate inhibit H_2_O_2_-induced oxidative stress in the human embryonic kidney [105]. Furthermore, oligophosphopeptides from phosvitin obtained after tryptic hydrolysis exhibited high antioxidant capacity in DPPH free-radical-scavenging tests [87] and in Caco-2 cells [86], showing a preventive effect against oxidation-induced DNA damage in vivo and preventing iron-mediated oxidative stress-related diseases, such as colorectal cancer [80].

## 5. Conclusions

Oxidative stress plays an important role in human health, and in food systems can increase the quality and the shelf life of products. Antioxidant compounds prevent oxidative and among them, natural antioxidants are considered safer for consumers than synthetic antioxidants, showing a growing interest in the last years. In addition to their high nutritional value, some chicken egg proteins and related ingredients (protein hydrolysates, peptides and amino acids) show antioxidant activity. There is extensive research on the evaluation of the antioxidant capacity of egg-derived hydrolysates and derived peptides. Many egg proteins such as ovalbumin, ovotransferrin, lysozyme, and cystatin from egg white or phosvitin from egg yolk are reported to have antioxidant properties by themselves. However bioactive peptides with increased antioxidant activity than proteins can be obtained after hydrolysis of egg proteins with proteolytic enzymes from animal, plant or bacterial origin as well as by chemical hydrolysis or gastrointestinal digestion. The whole egg white and yolk or a single protein are usually used as starting material to produce bioactive peptides and modification of egg proteins previously to the hydrolysis process using high-intensity pulsed electric field, sonication, heat and high-pressure treatments are used to increase radical scavenging properties. The sequences of several antioxidant peptides derived from the egg have been identified and their properties have been evaluated in chemical in vitro assays, cell cultures and animal models. However, a small number of pure peptides have been investigated at a cellular level and even less in in vivo systems, and moreover, there is a lack of digestibility and bioavailability studies as well as clinical trials. Therefore, further research is needed to recommend antioxidant egg-derived peptides for preventive and therapeutic treatments of both healthy subjects and patients with diseases related to stress oxidative. In addition, studies on the safety and quality of foods containing antioxidant peptides are also needed.

## Figures and Tables

**Table 1:** Sequences of antioxidant peptides obtained by hydrolysis of chicken egg white proteins.

Peptide Sequence	Protein of Origin	Starting Material	Enzymes	Antioxidant Assay	References
RVPSLM	OVT	EW	Alcalase	DPPH	[51]
TPSPR					
DLQGK					
AGLAPY					
RVPSL					
IRW	OVT	OVT	Thermolysin Pepsin	ORAC	[52]
LKP					
WNIP	OVT	OVT	Thermolysin	ORAC	[11]
GWNI					
YAEERYPIL	OVA	EW	Pepsin	DPPH, LP	[49]
SALAM					
YQIGL					
YRGGLEPINF					
DHPFLF	OVA	EW	Alcalase	DPPH	[51]
HAEIN					
QIGLF					
AEERYP	OVA	EW	Protease P	ORAC	[1]
AEERYP					
DEDTQAMP					
NTDGSTDYGILQINSR	LZ	LZ	Papain Trypsin	DPPH	[53]
RGY	LZ	LZ	Pepsin Trypsin α-chymotrypsin	RP, LP	[54]
WIR					
VAW					
VAWRNRCKGTD	LZ	LZ	Pepsin	ORAC, TBARS in Zebrafish larvae	[55]
IRGCRL					
WIRGCRL					
AWIRGCRL					
WRNRCKGTD					
WNWAD	OM	EW	Pepsin	ORAC, HEK-293 cells	[56]
PVDENDEG	CY	EW	Protease P	ORAC	[1]
HANENIF	EW	EW	Alcalase	DPPH	[51]
VKELY					
TNGIIR					
HTKE	EW	EW	Alcalase	RP, DPPH, ABTS, ORAC	[57]
FFGFN					
MPDAH					
DHTKE					
VYLPR	EW	EW	Alcalase	ORAC, ABTS, H2O2-induced oxidative damage on HEK-293	[44]
EVYLPR					
VEVYLPR					
VVEVYLPR					
YLGAK	EW	EW	Papain		[58]
GGLEPINFN					

OVT: ovotransferrin; EW: egg white; OVA: Ovalbumin; LZ: Lysozyme; OM: Ovomucoid; CY: Cystatin; DPPH: 2,2-difenil-1-picrylhydrazyl radical- scavenging activity assay; ORAC: Oxygen radical absorbance assay; RP: Reducing power assay; ABTS: 2,2’-azino-bis(3-ethylbenzothiazoline-6-sulfonic acid) radical-scavenging activity assay; TBARS: Thiobarbituric acid reactive substances; LP: Lipid peroxidation inhibition assay.

**Table 2:** Hydrolysis conditions applied to obtain antioxidant egg white hydrolysates using pepsin at pH 2.

Starting Material	Pre-Treatment	Pepsin Activity	Enzyme to Substrate Ratio	T (°C)	Time	Stop Conditions	References
Dissolved EW (100 mg/mL)	-	10,000 U/mg	1/100 (w/w)	37	3 h	pH 7	[49,61]
Pasteurized EW	-	3000 U/mg	2:100 (w/w)	38	8 h	pH 7	[62]
Freeze-dried EW	90 °C, 10 min	9000 U/g	30 g/L	37	5 h	85 °C, 30 min	[63]
EW powder 5.56%	90 °C, 10 min	NS	1.58% (w/w)	37	1 h	90 °C, 10 min	[12]
Liquid EW in water (1/4)	95 °C, 10 min	NS	0.4% (w/v)	37	1 h	Heat (NS)	[43]
Pasteurized EW	-	3000 U/mg	2:100 (w/w)	38	48 h	95 °C, 15 min	[64]

T: temperature; EW: egg white; NS: do not specify.
